# Periprosthetic Fluid Analysis in the Diagnosis of Breast Implant Infections Using Cell Count and Differential

**DOI:** 10.1093/asjof/ojaa028

**Published:** 2020-06-12

**Authors:** Christopher N Stewart, Bill B Liu, Eugene E Zheng, Sue-Mi C Tuttle

## Abstract

**Background:**

One of the most devastating complications following implant-based breast reconstruction is periprosthetic infection. Making a prompt and accurate diagnosis has been a challenge as plastic surgeons are limited by nonspecific systemic markers of infection, clinical examination findings, or imaging modalities.

**Objectives:**

The aim of this study is to evaluate the use of periprosthetic fluid using cell count and differential as an aid in the diagnosis of infection.

**Methods:**

This is a retrospective chart review. The authors selected patients who underwent breast reconstruction and had periprosthetic fluid analysis during the previous 10 years based on CPT 89051 (cell count and differential, body fluid). Only patients with clinical concerns for infection were included (cellulitis, fever, etc.); all others were excluded.

**Results:**

A total of 54 samples were included in the study. Twenty-seven samples were associated with periprosthetic breast infections based on positive cultures or intraoperative findings consistent with infection. On fluid analysis, those with infection had a significantly higher neutrophil percentage (84.2% vs 19.3%, *P* < 0.0001). A cutoff value of 77% neutrophils had a sensitivity of 89% and a specificity of 93% in diagnosing infection. Delayed treatment in patients with high neutrophil percentage was associated with poorer outcomes. Lastly, there was a strong correlation between higher neutrophil percentage and increased rate of capsular contracture.

**Conclusions:**

Early and accurate diagnosis of periprosthetic breast infections can lead to earlier treatment and potentially improved the outcomes. Aspiration and analysis of periprosthetic fluid for neutrophil percentage can be a reliable method to guide clinical decision making.

**Level of Evidence: 3:**

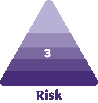

The incidence of reconstructive and cosmetic breast surgery has increased throughout the early 21st century and remains one of the most common procedures within plastic surgery.^1,2^ With the recent concerns surrounding breast implant-associated anaplastic large cell lymphoma, breast implant complications of all types have come into the national spotlight. Infectious complications continue to be a major concern, especially in the reconstructive patient. Rates of infection in these patients have been reported anywhere from 1% to 35%, and there is a clear association with patient comorbidities such as smoking, obesity, and prior radiation.^3^ Unfortunately, infections following breast implant placement often present a diagnostic dilemma. These infections are typically thought of as either being superficial or deep. Superficial infections such as cellulitis may resolve with nonoperative interventions, while deep infections that involve the periprosthetic generally require operative intervention. Unfortunately, classic clinical signs such as erythema, swelling, and fever, or laboratory values such as white blood cell (WBC) count and C-reactive protein (CRP) do not reliably distinguish between the two. As a result, appropriate treatment of these infections is often delayed while awaiting culture results or clinical change in appearance.

In a landmark paper in 2004, Trampuz et al^4^ showed synovial fluid cell count and differential could be reliably used to diagnose infection in patients who had previously undergone total knee arthroplasty using cutoff values for a total cell count of 1.7 × 10^3^/µL and 65% neutrophils (polymorphonucleocytes [PMNs]). Since that time, it has become standard of care for orthopedic surgeons to use synovial cell count and differential of aspirated fluid to guide the diagnosis of knee implant infections.^5^ Furthermore, a meta-analysis also showed that using synovial cell count and differential is a reliable way to diagnose both knee and hip arthroplasty infections. The reviewed studies used a total cell count cutoff anywhere from 2500 to 50,000 cells/µL and 60% to 89% neutrophils as highly suggestive of infection.^6^

Patients who present with periprosthetic fluid collections and concern for infection often have this fluid aspirated and sent for bacterial culture and gram stain. Although specific microbial information is essential for definitive treatment, these results often take numerous days, may be unreliable due to concomitant antibiotic use, and often return after the clinical course has already been determined. In this study, our aim is to evaluate the use of cell count and differential from periprosthetic fluid to establish a prompt diagnosis of periprosthetic breast infection in order to guide appropriate clinical therapy and improve patient outcomes.

## METHODS

Approval was obtained from the HealthPartners Institute Institutional Review Board prior to beginning this study.

### Patient Selection

All patients with a history of implant-based breast reconstruction (permanent prosthesis or tissue expander) who had periprosthetic fluid analysis in the past 4 years (December 2014 to December 2018) for cell count and differential of body fluid (CPT code 89051), were screened using EPIC software (EPIC systems, Verona, WI). Only samples obtained from the periprosthetic space for clinical concern for infection (fever, cellulitis, etc) were included. Drain fluid was not sampled. Patients with a diagnosis of implant exposure or hematoma were excluded from the study. Patients were followed clinically per each staff’s routine. All patients were treated as per accepted standards of care and informed consent was obtained prior to any intervention. Capsular contracture was diagnosed clinically and classified using Baker grade. Patient demographics, laboratory, and clinical data were extracted from the electronic medical records. 

### Fluid Analysis

Periprosthetic fluid samples were obtained aseptically either in the clinical setting with direct aspiration over expansion port, under ultrasound guidance for permanent prosthesis, or intraoperatively with direct aspiration of fluid from the periprosthetic space. The samples were then analyzed by microscopic examination to determine nucleated cell count and differential. The samples were also sent for aerobic, anaerobic, and fungal cultures.

### Statistical Analysis

Numeric variables with normal distribution were compared using a 2-sample *t*-test or 1-way analysis of variance (ANOVA). Numeric variables with non-normal distributions (PMN%, WBC, CRP, erythrocyte sedimentation rate [ESR]) were analyzed using Mann–Whitney test. Nucleated cell count was log-transformed for statistical analysis due to asymmetric distribution and unequal variance. Categorical variables were compared using the likelihood-ratio test. Fisher’s exact test was used in contingency table analysis. Cutoff values for cell counts were determined using receiver operating characteristic (ROC) analysis. Sensitivity, specificity, and positive and negative predictive values were calculated using contingency tables. *P* < 0.05 (2-sided) was considered statistically significant. JMP Pro (Cary, NC, version 14.0.0) was used for all statistical and graphical analysis. 

## RESULTS

A total of 54 fluid samples from 44 patients were included in the study. Multiple samples from the same patient represent distinct reconstructions (eg, different laterality or phase of reconstruction). All patients were female. The mean age of our patient cohort is 52 years (range, 30-81 years). Mean BMI is 29.8 (range, 18-46.1). The mean length of follow-up from the time of fluid aspiration is 18 months (range, 0-42 months). Periprosthetic breast infection was diagnosed if the associated culture was positive or if there were intraoperative findings suggestive of infection in the periprosthetic space (purulence, cloudy fluid, significant fibrinous debris, etc.). The mean time from initial implant placement to sampling is 68 days and the median is 46 days. Based on this information, 27 samples were classified into the “periprosthetic infection” (PI) group and 27 into the “no periprosthetic infection” (NPI) group (Table 1). Seven samples were associated with permanent prosthesis and 47 associated with tissue expanders. Acellular dermal matrix was used in 85% of all cases. There were no statistical differences between baseline characteristics such as age, BMI, rate of diabetes, tobacco use, and history of radiation or chemotherapy in PI and NPI groups except for the need for surgical intervention within 90 days of presentation (96% vs 15%, *P* < 0.0001).

**Table 1.** Patient Characteristics

	Periprosthetic infection (PI), *N* = 27	No periprosthetic infection (NPI), *N* = 27	*P*
Age (30-81 years)	50.4 (2.2)	53.9 (2.4)	0.29
BMI (18-46.1 kg/m^2^)	30.4 (1.1)	29.2 (1.3)	0.49
Diabetes	1 (3.7%)	2 (7.4%)	0.55
Current tobacco use	3 (11.1%)	2 (7.4%)	0.64
History of radiation	4 (14.8%)	5 (18.5%)	0.71
History of chemotherapy	16 (59.3%)	15 (55.6%)	0.78
Tissue expander	23 (85.2%)	24 (88.9%)	0.68
Permanent prosthesis	4 (14.8%)	3 (11.1%)	0.68
ADM use	21 (77.8%)	25 (92.6%)	0.12
Surgical intervention	26 (96.3%)	4 (14.8%)	<0.0001*

Mean, range, and stand errors are shown for age and BMI. The number of patients and percentages is shown for other variables. ADM, acellular dermal matrix; BMI, body mass index. *Statistically significant, *P* <0.05.

**Figure 1.**
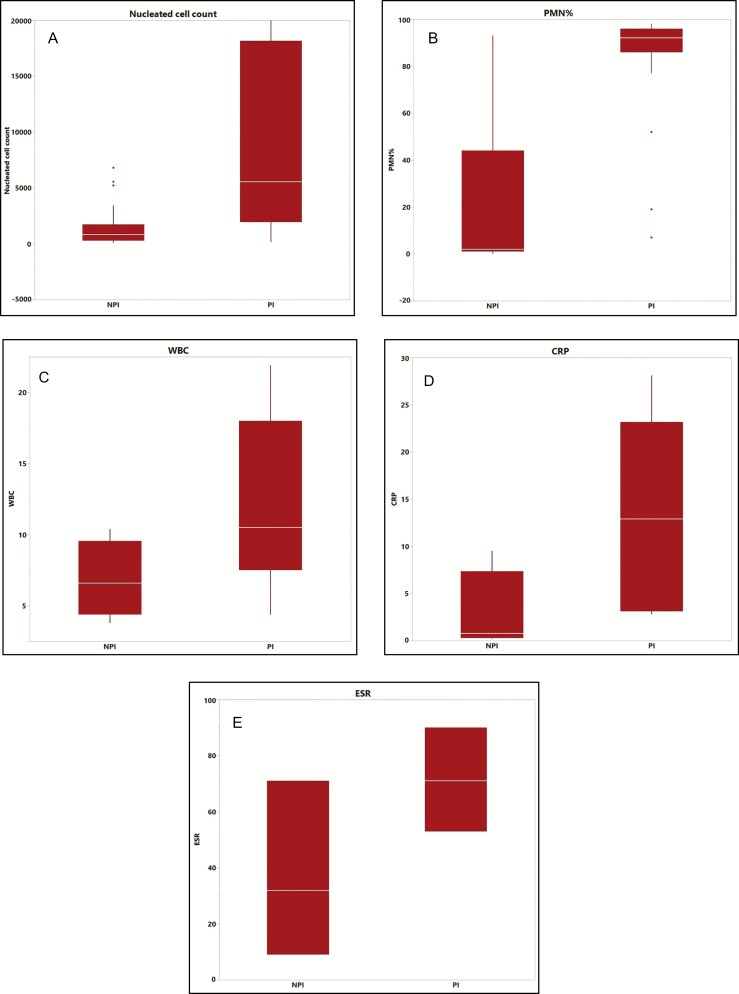
Box plots of periprosthetic fluid cell count and differential vs plasma levels of traditional inflammatory markers. PI, Periprosthetic Infection; NPI, No periprosthetic infection. (A) Total nucleated cell count. (B) Percentage of polymorphonuclear neutrophils, PMN%. (C) White blood cell count, WBC. (D) C-reactive protein, CRP. (E) Erythrocyte sedimentation rate, ESR. Note the greater separation of the 2 groups with fluid analysis markers compared with traditional inflammatory markers.

Cell count and differential along with plasma levels of traditional inflammatory markers for both groups were compared, and the details are shown in Table 2 and Figure 1. Nucleated cell count was significantly higher in the PI group compared with the NPI group (25,141 vs 1468/µL, *P* < 0.0001). Similarly, neutrophil percentage was significantly higher in the PI group (84.2% vs 19.3%, *P* < 0.0001). In contrast, traditional inflammatory markers performed much worse in distinguishing a difference between the 2 groups, with only CRP reaching statistical significance at *P* = 0.043.

**Table 2.** Periprosthetic Cell Count and Differential and Traditional Inflammatory Markers

	Periprosthetic infection (PI), *N* = 27	No periprosthetic infection (NPI), *N* = 27	*P*
Nucleated cell count	25,141.4 (10,676.3)	1,468.4 (345.5)	<0.0001*
PMN%	84.2 (4.4)	19.3 (5.5)	<0.0001*
WBC	11.6 (1.5)	6.9 (1.4)	0.099
CRP	13.6 (4.1)	2.8 (2.2)	0.043*
ESR	71.3 (10.7)	37.3 (18.1)	0.268
Positive culture	19/27	0/27	<0.0001*

Results shown with mean and standard error. CRP, C-reactive protein; ESR, erythrocyte sedimentation rate; PMN%, neutrophil percentage; WBC, white blood cell. *Statistically significant, *P* <0.05.

Using ROC analysis, we were able to determine an optimal PMN% cutoff value of 77%. This value has a sensitivity of 89% and a specificity of 93% in diagnosing periprosthetic breast infection. This also yields a positive predictive value of 92%. Various cutoff values and the associated diagnostic performance are also included (Table 3).

**Table 3.** Comparison of Various Cutoff Values for Neutrophil Percentage (PMN%)

PMN% cutoff value	Sensitivity	Specificity	Positive predictive value	Negative predictive value
85	0.81	0.96	0.96	0.84
81	0.81	0.93	0.92	0.83
79	0.85	0.93	0.92	0.86
77	0.89	0.93	0.92	0.89
69	0.89	0.89	0.89	0.89
55	0.89	0.85	0.86	0.88
52	0.93	0.85	0.86	0.92

PMN%, neutrophil percentage.

**Figure 2.**
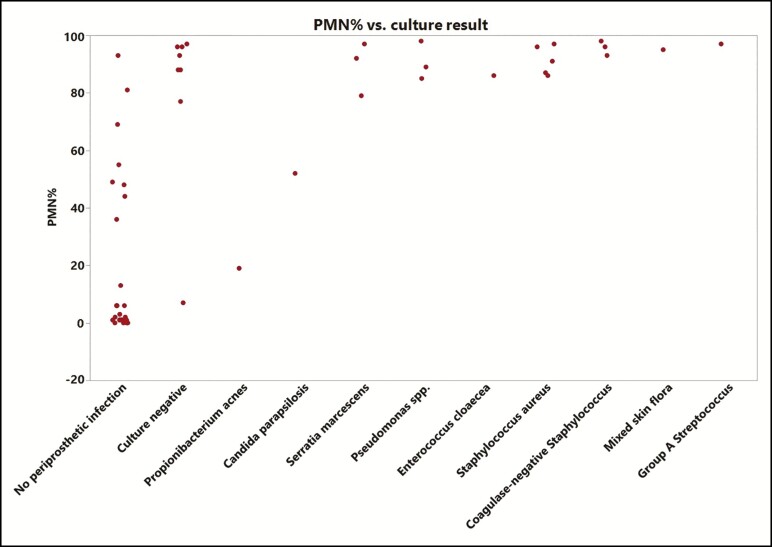
Neutrophil percentage (PMN%) by associated culture results.

The most common culture result for the periprosthetic infection group was culture negative (30%). Causative microorganisms were identified in 70% of samples, with *Staphylococcus aureus* being the most common pathogen (26% of samples), followed by coagulase-negative *Staphylococcus*, *Pseudomonas* spp., and *Serratia marcescens* (Figure 2). Out of the 54 samples, 34 were receiving antibiotics at the time of aspiration. For the culture-negative samples, 100% were on antibiotics at the time of aspiration, compared with 74% for the culture-positive samples. The PMN% associated with each culture result is as follows: *Group A Streptococcus* (97%), coagulase-negative *Staphylococcus* (96%), mixed skin flora (95%), *S. aureus* (91%), *Pseudomonas aeruginosa* (91%), *Propionibacterium acnes* (19%), and *Candida parapsilosis* (52%). 

**Figure 3.**
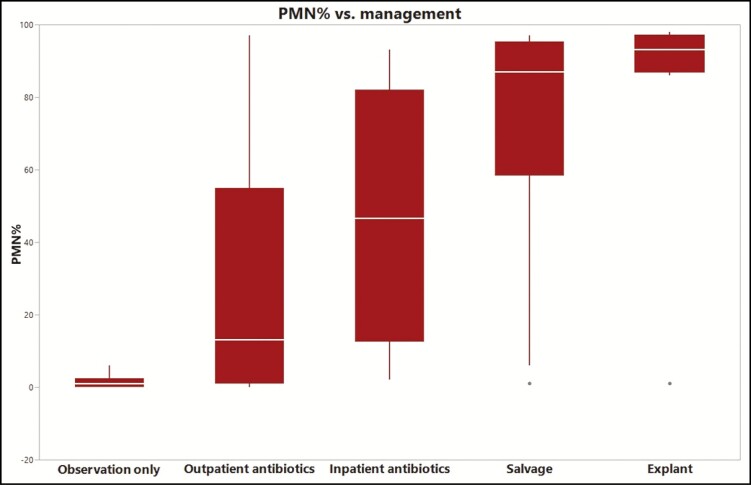
Neutrophil percentage (PMN%) vs associated outcomes.

Neutrophil percentage was then correlated with patient outcome. Patients were identified as nonoperative or operative based on whether they had surgical interventions within 90 days of the fluid analysis. The nonoperative group was then subclassified into outpatient vs inpatient management based on admissions related to the breast implant infection within 90 days of fluid analysis. The operative group was also subclassified into “salvage” if the implant was replaced and “explant” if it was not. Average neutrophil percentage (PMN%) for patients who received operative vs nonoperative management was 75% and 22%, respectively (*P* < 0.0001). The explant subgroup trended toward the highest PMN% of all (Figure 3).

**Figure 4.**
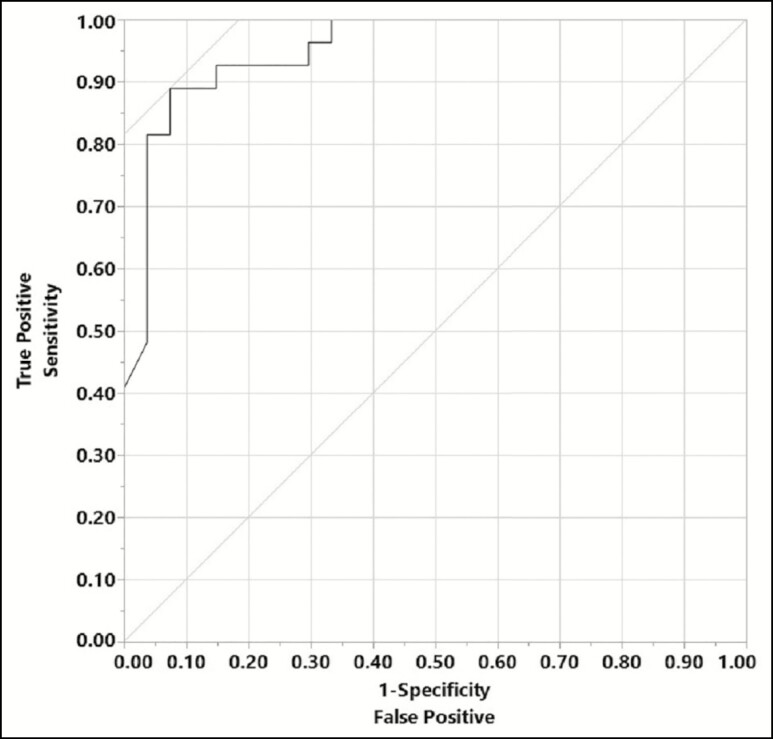
Receiver operating characteristic curve for neutrophil percentage (PMN%). Area under curve (AOC) = 0.95.

**Figure 5.**
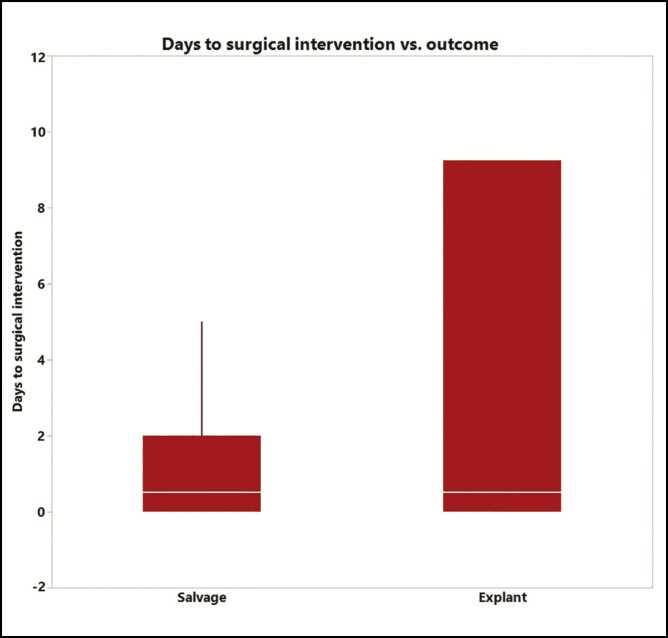
Time (in days) between fluid sampling and outcome (salvage vs explant).

**Figure 6.**
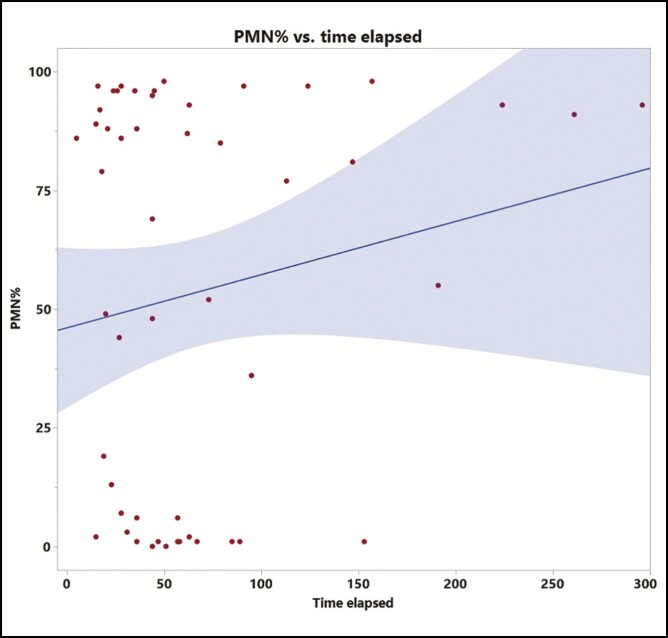
Bivariate analysis of time elapsed (in days) between initial implant placement and PMN% at the time of fluid sampling. Line of best fit shown with correlation coefficient, *r*^2^ = 0.029, indicating a limited relationship between these variables.

Receiver operating characteristic analysis was used to determine the optimal cutoff values for diagnosing periprosthetic breast infection. These were found to be 1777/µL for nucleated cell count (sensitivity 81% and specificity 78%) and 77% for neutrophil percentage (sensitivity 89% and specificity 93%). The area under curve for PMN% is 0.95 (Figure 4). Time to surgical intervention from sample collection was 1 ± 1.5 days for the salvage group and 7.2 ± 13.9 days for the explant group, *P* = 0.06 (Figure 5). The time between initial implant placement and PMN% at the time of fluid sampling did not show a significant relationship on bivariate analysis (*r*^2^ = 0.029, Figure 6). Median inpatient length of stay for antibiotics only, salvage, and explant groups were 3, 1.5, and 2 days, respectively.

Patients with follow-up longer than 12 months were included in capsular contracture analysis. Overall, the capsular contracture rate from our data is 20% from 45 samples. For nonoperatively managed patients, those with PMN% greater than our proposed cutoff of 77% had a capsular contracture rate of 100% (2 out of 2) compared with 11% (2 out of 18) for those with PMN% less than 77%, *P* = 0.006 (Table 4). For surgically managed patients, PMN% was not a statistically significant predictor of capsular contracture rate (rate of 26% vs 0% for PMN% greater and less than 77% respectively, *P* = 0.078).

**Table 4.** Capsular Contracture Rate

	PMN% < 77	PMN% > 77	*P*
Nonoperative (*N* = 20)	11.1% (2/18)	100% (2/2)	0.0063*
Operative (*N* = 25)	0% (0/6)	26.3% (5/19)	0.078

Capsular contracture is defined as positive for grade 2-4 at 1 year or longer follow-up. Nine patients did not have documentations of capsular contraction but were excluded for this analysis due to a follow-up period of less than 1 year. PMN%, neutrophil percentage. *Statistically significant, *P* <0.05.

## DISCUSSION

Periprosthetic breast infections often have devastating consequences. Early diagnosis and treatment are the mainstay of reconstruction salvage. Currently, surgeons rely on clinical examination and traditional inflammatory markers such as WBC count or CRP to determine the need for surgical intervention. However, these measures do not reliably distinguish true periprosthetic infections from superficial infections or other conditions such as reactive erythema and seroma. Periprosthetic fluid sampling is a relatively noninvasive diagnostic tool that can be safely and comfortably performed in the clinic setting, especially for patients with tissue expanders.^7^ The fluid can then be used to determine whether the source of infection is truly in the periprosthetic space. This allows for earlier surgical intervention and maximizes the chance of reconstructive salvage while avoiding unnecessary procedures or prolonged hospital stays.

Over the past 15 years, there have been numerous publications in the orthopedic literature relating to the accurate and prompt diagnosis of periprosthetic infections for arthroplasty patients, often in the setting of minor clinical manifestations. In 2004, Trampuz et al were the first to report the use of periprosthetic fluid in the diagnosis of implant infections. In their landmark study, they found a nucleated cell count greater than 1700/µL to be 94% sensitive and a neutrophil percentage greater than 65% to be 97% sensitive in diagnosing septic failure.^4^ In 2010, Bedair et al^8^ reviewed over 11,000 implants and found that those with positive cultures or purulence at the time of operation had a significantly higher percentage of neutrophils on fluid analysis than those without infection (89.6% vs 76.9%) and that the optimal cutoff value was 89% PMNs. Following this in 2014, Yi et al^9^ reviewed 6000 more cases and found similar results with a PMN% cutoff value of 89% suggesting infection. In 2011, this practice was published as a clinical guideline for orthopedic surgeons and 2 separate meta-analyses that followed supported the use of cell count and differential in the early diagnosis of periprosthetic infection.^5,6,10^

Using the Trampuz paper as a parallel framework, we hope that our study will further develop the postoperative management of breast surgery patients and aid surgeons in clinical decision making for breast surgery complications. In this study, we compared 27 periprosthetic fluid samples that were associated with periprosthetic breast infections to 27 samples that were not. Our results are consistent with published data regarding different types of periprosthetic infections (Table 2). In samples that were associated with periprosthetic infections, we found total nucleated cell count and neutrophil percentage (PMN%) to be higher than those that were not (25,141 vs 1468/µL and 84.2% vs 19.3%, respectively). We also found that these markers significantly outperformed traditional markers of infection such as WBC, ESR, and CRP in diagnosing periprosthetic breast infections, as had been described in previous literature^10^ (Figure 1). In addition, traditional inflammatory markers do not aid in the diagnosis when there are other potential sources of infection. For instance, one of our patients had a concurrent intra-abdominal infection, which renders the interpretation of elevated systemic inflammatory markers difficult. However, nucleated cell count of 2.6 × 10^5^/µL and PMN% of 97% localized the infection to the periprosthetic space, which was then noted to have purulence intraoperatively and yielded positive culture results.

Using our data, we found that neutrophil percentage (PMN%) was the variable that most reliably diagnosed periprosthetic breast infections. Time elapsed from initial implant placement was not related to PMN% at the time of sampling (Figure 6). Nucleated cell count, despite reaching statistical significance (*P* < 0.0001), was deemed not to be the ideal diagnostic criteria due to greater overlap of values between the 2 groups, resulting in lower optimized sensitivity and specificity (81% and 78%, respectively for cutoff of 1777/µL). One possible reason for this discrepancy between our data and the established orthopedic literature is that periprosthetic volume is significantly more variable in the breast reconstruction setting than the relatively fixed volume seen in arthroplasty, resulting in significant variation of the cell count in the periprosthetic fluid. However, we postulate that because the neutrophil percentage is agnostic to the absolute number of cells in the fluid, it is likely not affected by this caveat.

In 2013, Reish et al^11^ reviewed the outcomes of periprosthetic breast implant infections and the predictors of salvage vs explant. Their study showed that 37% of implants were able to be salvaged and that predictors of failure included smoking, chemo-radiation, and methicillin-resistant *Staphylococcus aureus* infection. In our study, we were able to show a strong correlation between PMN% and the likelihood of salvage using antibiotic therapy alone, breast pocket irrigation and implant exchange, and explantation (Figure 3). Although our study was not designed to conclude a causal relationship between surgical intervention for suspected periprosthetic infections and the rate of capsular contracture, we found that patients with a high PMN% who were managed nonoperatively had significantly increased rate of capsular contracture (100% vs 11.1%, Table 4). Interestingly, the one patient who was managed nonoperatively despite positive culture result due to significant clinical improvement on antibiotics alone had a PMN% of 97% at the time of presentation and went on to develop capsular contracture. This is consistent with studies suggesting that overactive inflammatory response, low-level chronic inflammation, or the presence of biofilm and subclinical infection can lead to capsular contracture.^12^ The goal of this study was not to evaluate the role of biofilm in breast implant infection and unfortunately with only a single patient a causal association cannot be made from this finding.

There have been previous studies looking at the microbiology of breast implant infections. The most common causative organisms are gram-positive and consist of *Staphylococcus* and *Streptococcus* species.^13^ We also found a similar distribution of causative organisms in our data set and we were also able to show the correlation of PMN% with the various pathogens. Although not reaching statistical significance, there was a trend of higher PMN% associated with more virulent strains of bacteria such as *Staphylococcus* and *Pseudomonas* species. Eight patients with periprosthetic breast infection were culture negative, suggesting that antibiotics are having an influence on culture results. There were no positive cultures in the non-infected group. However, as we show in Table 2, there is still a statistically significant difference in PMN% between the infection and non-infection groups. Regardless of antibiotic treatment in our study, when we observe a decrease in PMN%, we see a statistically significant increase in salvage (Figure 3). Further, when running a 1-way ANOVA for PMN% and concurrent use of antibiotics, a higher PMN% is associated with concurrent use of antibiotics at the time of the sample (mean of 65% for those on antibiotics and 29% for those not on antibiotics, *P* = 0.0013). However, this is likely correlational instead of causal as patients with more severe infection clinically are more likely to have been placed on antibiotics. From our data, it does not appear that antibiotic use at the time of aspiration results in an artificially lower PMN%.

In this study, we represent the first to use an objective laboratory test to identify periprosthetic breast infections. Unfortunately, there is a variable presentation of these patients and thus no “gold standard” of treatment. Typical treatment patterns may include outpatient antibiotic therapy, admission to the hospital with IV antibiotics, or urgent operative intervention. Some providers may start antibiotics and follow the patient clinically prior to decision making. In these methods, by the time any fluid is aspirated, the patients have been initiated on antibiotics, and culture results are unreliable. By sampling periprosthetic fluid and analyzing for neutrophil percentage, we believe that one can obtain an accurate and early diagnosis to potentially avoid delays in surgical intervention. Based on our results, PMN% percentage can also guide the decision to washout and replace an implant vs explantation. Regardless of variable clinical examination, we also noted a correlation between days until surgical intervention and rates of salvage vs explanation, suggesting that any delay may lead to worse outcomes.

Our data did not include an economic analysis; however, one can postulate that earlier and accurate diagnosis can lead to reduced costs. Olsen et al^14^ reviewed the cost of surgical site infections following mastectomy with breast reconstruction and, after controlling for procedure type, found an increase of over $4000 in the infection group. They also found that these patients had an increased length of stay on average of just over 4 days. In our study, the average length of stay for the PI group was 2.6 days vs 0.5 days for the NPI group. Median inpatient length of stay in days for antibiotics only, salvage, and explant groups were 3, 1.5, and 2 days, respectively. While the differences between these groups did not reach statistical significance, there is a trend toward a shorter length of stay for early operative intervention when indicated, which could have a large economic impact.

Because textured implants are known to harbor more surface bacteria than smooth implants, theoretically this could have an impact on the fluid analysis results. Although our study is not powered to fully assess the significance of implant texturing, we did not notice any difference in fluid analysis results, rates of infection, the likelihood of surgical intervention, or capsular contracture rates between smooth and textured implants.

Our current study is not without limitations. First, it is a retrospective analysis of patients that had previously undergone intervention based on clinical factors. Cell count and differential were known at the time but were not necessarily used to guide clinical decision making. This is also a single-center study with most patients from 2 surgeons. Anytime post hoc analysis is performed, one should use caution in drawing conclusions. However, the trend in our data is strong and despite having a relatively small sample size, our analysis easily reaches both clinical and statistical significance. Additional prospective studies are needed to explore the significance of PMN% in distinguishing virulent vs non-virulent strains of bacteria or other mimicking conditions such as red-breast syndrome. This would also include sampling and comparing fluid to patients without clinical evidence of infection. Further, as more advanced diagnostic methods, such as 16s rRNA sampling, become readily available, we may directly compare our data to these results and evaluate if this tool can be further refined to aid in patient selection for successful immediate implant salvage vs explant based on the breast microbiome.

## CONCLUSIONS

The prompt diagnosis of periprosthetic breast infection can be challenging and stressful for both plastic surgeons and patients alike. Without hard indications for surgical intervention, the surgeon is often left with a “wait and see” approach. This could potentially result in decreased salvage rate or overly aggressive surgical intervention leading to unnecessary procedures and prolonged hospitalization. By using periprosthetic fluid analysis and PMN%, one can potentially come to a prompt and reliable diagnosis in order to guide treatment and improve patient outcomes.

## References

[1] The American Society for Aesthetic Plastic Surgery. Cosmetic Surgery National Data Bank: Statistics 2018. Aesthet Surg J. 2019;39(Suppl_4):1-27. 10.1093/asj/sjz164 31226205

[2] Agency for Healthcare Research and Quality. Breast reconstruction surgeries after mastectomies increased more than 60 percent from 2009 to 2014. http://www.ahrq.gov/news/newsroom/press-releases/breastreconstruct1010.html. Accessed April 4, 2020.

[3] Washer LL , Gutowski K. Breast implant infections. Infect Dis Clin North Am. 2012;26(1):111-125. 22284379 10.1016/j.idc.2011.09.003

[4] Trampuz A , Hanssen AD, Osmon DR, Mandrekar J, Steckelberg JM, Patel R. Synovial fluid leukocyte count and differential for the diagnosis of prosthetic knee infection. Am J Med. 2004;117(8):556-562. 15465503 10.1016/j.amjmed.2004.06.022

[5] Della Valle C , Parvizi J, Bauer TW, et al.; American Academy of Orthopaedic Surgeons. American Academy of Orthopaedic Surgeons clinical practice guideline on: the diagnosis of periprosthetic joint infections of the hip and knee. J Bone Joint Surg Am. 2011;93(14):1355-1357. 21792503 10.2106/JBJS.9314ebo

[6] Qu X , Zhai Z, Liu X, et al. Evaluation of white cell count and differential in synovial fluid for diagnosing infections after total hip or knee arthroplasty. PLoS One. 2014;9(1):e84751. 24416276 10.1371/journal.pone.0084751 PMC3885622

[7] Moyer KE , Potochny JD. Technique for seroma drainage in implant-based breast reconstruction. J Plast Reconstr Aesthet Surg. 2012;65(12):1614-1617. 22770571 10.1016/j.bjps.2012.06.016

[8] Bedair H , Ting N, Jacovides C, et al. The Mark Coventry Award: diagnosis of early postoperative TKA infection using synovial fluid analysis. Clin Orthop Relat Res. 2011;469(1):34-40. 20585914 10.1007/s11999-010-1433-2 PMC3008895

[9] Yi PH , Cross MB, Moric M, Sporer SM, Berger RA, Della Valle CJ. The 2013 Frank Stinchfield Award: diagnosis of infection in the early postoperative period after total hip arthroplasty. Clin Orthop Relat Res. 2014;472(2):424-429. 23884798 10.1007/s11999-013-3089-1 PMC3890203

[10] Lee YS , Koo KH, Kim HJ, et al. Synovial fluid biomarkers for the diagnosis of periprosthetic joint infection: a systematic review and meta-analysis. J Bone Joint Surg Am. 2017;99(24):2077-2084. 29257013 10.2106/JBJS.17.00123

[11] Reish RG , Damjanovic B, Austen WG Jr, et al. Infection following implant-based reconstruction in 1952 consecutive breast reconstructions: salvage rates and predictors of success. Plast Reconstr Surg. 2013;131(6): 1223-1230. 23714788 10.1097/PRS.0b013e31828bd377

[12] Ajdic D , Zoghbi Y, Gerth D, Panthaki ZJ, Thaller S. The relationship of bacterial biofilms and capsular contracture in breast implants. Aesthet Surg J. 2016;36(3):297-309. 26843099 10.1093/asj/sjv177 PMC5127460

[13] Franchelli S , Pesce M, Baldelli I, Marchese A, Santi P, De Maria A. Analysis of clinical management of infected breast implants and of factors associated to successful breast pocket salvage in infections occurring after breast reconstruction. Int J Infect Dis. 2018;71:67-72. 29660396 10.1016/j.ijid.2018.03.019

[14] Olsen MA , Chu-Ongsakul S, Brandt KE, Dietz JR, Mayfield J, Fraser VJ. Hospital-associated costs due to surgical site infection after breast surgery. Arch Surg. 2008;143(1):53-60; discussion 61. 18209153 10.1001/archsurg.2007.11

